# Modulation of Influenza A virus NS1 expression reveals prioritization of host response antagonism at single-cell resolution

**DOI:** 10.3389/fmicb.2023.1267078

**Published:** 2023-10-09

**Authors:** Qing Yang, Anna E. Elz, Maryline Panis, Ting Liu, Benjamin E. Nilsson-Payant, Daniel Blanco-Melo

**Affiliations:** ^1^Vaccine and Infectious Disease Division, Fred Hutchinson Cancer Center, Seattle, WA, United States; ^2^Innovation Laboratory, Fred Hutchinson Cancer Center, Seattle, WA, United States; ^3^Department of Microbiology, New York University, New York, NY, United States; ^4^TWINCORE Centre for Experimental and Clinical Infection Research, Institute of Experimental Virology, Hannover, Germany; ^5^Cluster of Excellence RESIST (EXC 2155), Hanover Medical School, Hanover, Germany; ^6^Herbold Computational Biology Program, Public Health Sciences Division, Fred Hutchinson Cancer Center, Seattle, WA, United States

**Keywords:** influenza, NS1, interferon, immune antagonism, innate immune response, unfolded protein response, single cell transcriptomics

## Abstract

Influenza A virus (IAV) is an important human respiratory pathogen that causes significant seasonal epidemics and potential devastating pandemics. As part of its life cycle, IAV encodes the multifunctional protein NS1, that, among many roles, prevents immune detection and limits interferon (IFN) production. As distinct host immune pathways exert different selective pressures against IAV, as replication progresses, we expect a prioritization in the host immune antagonism by NS1. In this work, we profiled bulk transcriptomic differences in a primary bronchial epithelial cell model facing IAV infections at distinct NS1 levels. We further demonstrated that, at single cell level, the intracellular amount of NS1 in-part shapes the heterogeneity of the host response. We found that modulation of NS1 levels reveal a ranking in its inhibitory roles: modest NS1 expression is sufficient to inhibit immune detection, and thus the expression of pro-inflammatory cytokines (including IFNs), but higher levels are required to inhibit IFN signaling and ISG expression. Lastly, inhibition of chaperones related to the unfolded protein response requires the highest amount of NS1, often associated with later stages of viral replication. This work demystifies some of the multiple functions ascribed to IAV NS1, highlighting the prioritization of NS1 in antagonizing the different pathways involved in the host response to IAV infection.

## Introduction

Upon viral infection, sensing of pathogen-associated molecular patterns (PAMPs) by intracellular pattern recognition receptors (PRRs) activates a signaling cascade that results in the production of type I and III interferons (IFN-I and IFN-III). After being secreted into the extracellular milieu, IFNs lead to the rapid induction of a potent antiviral response characterized by the expression of hundreds of IFN-stimulated genes (ISGs) in infected and neighboring cells (Grandvaux et al., 2002). These ISGs contribute to the establishment of the antiviral state by directly antagonizing the virus, driving the expression of secondary antiviral products and regulating the overall antiviral response (Schoggins, 2019). To establish successful infections, viruses must circumvent the host innate immunity by simultaneously evading detection and directly inhibiting host immune modulatory proteins.

Influenza A virus (IAV) is an important human respiratory pathogen that causes 3–5 million cases of severe illness each year (WHO, 2023), and under certain conditions, emerging IAV strains can cause serious pandemics (Shao et al., 2017). IAV has a segmented, single-stranded, negative-sense RNA genome, where each segment is associated with the viral RNA-dependent RNA polymerase and is wound around a double-helical nucleoprotein scaffold to form a viral ribonucleoprotein structure (Te Velthuis and Fodor, 2016). This structure shields the genome from immune detection and is responsible for the transcription and replication of the viral genome (Fodor and Te Velthuis, 2019). Besides evading immune detection, IAV also deploys multiple mechanisms to attenuate the host response, most of which have been ascribed to the viral protein NS1. Depending on the viral strain, NS1 is able to block viral recognition by PRRs, inhibit the activation of transcription factors required for IFN-I induction, suppress the antiviral effects of certain ISGs, and limit the processing and nuclear export of host mRNAs (including IFNs), among other pro-viral roles (Hale et al., 2008; Garcia-Sastre, 2011; Krug, 2015). With its numerous facets of host immune suppression, the accumulation of NS1 must be fine-tuned during IAV life cycle to accommodate viral transcription, packaging, and egress, during which different PAMPs are exposed to host detection or inhibition. Under this assumption, NS1 would further prioritize antagonism of different host immune pathways. However, how the relative amount of NS1 correlates to its role in innate immune suppression is poorly understood.

As NS1 is transcribed through alternative splicing of the IAV NS segment, which additionally encodes the nuclear export protein (NEP), it’s challenging to modulate NS1 amount independently without altering the level of NEP. While NS1 deletion viruses (IAV-ΔNS1) have been instrumental to understand the full extent of NS1 antagonism (Garcia-Sastre et al., 1998), it is not directly evident which host pathways would be more sensitive to this viral antagonist. So far, Chua et al. (2013) devised a system that uses intrinsic microRNA (miRNA) targeting to silencing NS1 while sparing NEP expression. However, they found silencing NS1 does not affect host antiviral response, and the authors attributed this to the limited immune capacity in immortalized systems. Follow-up single cell transcriptomic studies highlighted that viral NS1 expression levels in-part anti-correlates with host immune gene expression but did not define which host pathways are more impacted by varying NS1 levels (Russell et al., 2018; Ramos et al., 2019; Sun et al., 2020; Vicary et al., 2023).

We anticipate that host immune pathways that exert the highest selective pressure on IAV would be prioritized by NS1 (i.e., more susceptive to NS1 abundance). To better capture the relationship between IAV NS1 expression and the host antiviral response, we characterized the transcriptional landscape of primary bronchial epithelial cells upon infection with wild type and recombinant viruses with varying levels of NS1. We hence examined host response to wild type IAV infection at the single cell level and found the heterogeneous host immune state is heavily shaped by the intracellular NS1 levels. This work reveals the prioritization of host immune antagonism by NS1: lesser amount of NS1 is required to silence IFN production than to suppress ISG expression or the unfolded protein response (UPR). Together, our work highlights the adaptation of IAV host antagonism towards its replication needs.

## Materials and methods

### Cell culture

MDCK and MDCK-NS1-GFP (Kochs et al., 2009) cells were maintained at 37°C and 5% CO2 in Dulbecco’s Modified Eagle Medium (DMEM, Gibco^®^) supplemented with 10% Fetal Bovine Serum (FBS, Corning^®^) as described elsewhere (Chua et al., 2013). Normal human bronchial epithelial (NHBE) cells (Lonza, CC-2540 Lot# 580580) were isolated from a 79 years-old Caucasian female and were maintained in bronchial epithelial growth media (Lonza, CC-3171) supplemented with BEGM SingleQuots as per the manufacturer’s instructions (Lonza, CC-4175) at 37°C and 5% CO2.

### Viruses

Influenza A/Puerto Rico/8/34 (H1N1) viruses (NCBI:txid183764) were grown for 48 h in MDCK cells (for IAV-WT) or MDCK-NS1-GFP cells [for IAV-NS1-T and IAV-ΔNS1 (Garcia-Sastre et al., 1998)] at an MOI of 0.05 in DMEM supplemented with 0.3% BSA (MP Biomedicals^®^) and 1 μg/mL TPCK-trypsin (Sigma-Aldrich^®^). The IAV-NS1-T virus was derived from a NS1-NEP split influenza A/Puerto Rico/8/34 (H1N1) virus and was constructed following a previously described strategy where tandem target sites for miRNA-30 were inserted into the 3′ UTR of the NS1 gene (Chua et al., 2013). Infectious titers of influenza A viruses were determined by plaque assays in MDCK or MDCK-NS1-GFP cells. Infections with wild-type or mutant IAV were performed at the indicated MOIs for 1 h at 37°C in DMEM supplemented with 0.3% BSA and 1 μg/mL TPCK-trypsin before incubation for the indicated hours post infection (hpi) at 37°C. For infection of NHBE cells with IAV, cells were washed with HEPES buffered saline solution (Lonza, CC-5024) after initial adsorption of the virus and supplemented with fresh media for the indicated amount of time at 37°C. NHBE cells were also treated with 100 units of human IFN-β for 4, 6 or 12 h, as indicated.

### Western blot

Cells were lysed in NP40 lysis buffer containing 1× Complete Protease Inhibitor Cocktail (Roche^®^) and 1× Phenylmethylsulfonyl fluoride (Sigma Aldrich^®^) and cleared from the insoluble fraction by centrifugation at 17,000 × *g* for 5 min at 4°C. Samples were separated by SDS-PAGE and transferred onto nitrocellulose membranes. Proteins were detected using mouse monoclonal anti-actin (Thermo Scientific, MS-1295), mouse monoclonal anti-tubulin (Developmental Studies Hybridoma Bank JLA20), rabbit monoclonal anti-IFIT1 (Cell Signaling, D2X9Z), rabbit polyclonal anti-GFP (Abcam, ab290), rabbit polyclonal anti-MX1 (Abcam, ab207414), mouse monoclonal anti-IAV NP (Center for Therapeutic Antibody Discovery at the Icahn School of Medicine at Mount Sinai, clone HT103), mouse monoclonal anti-IAV NS1 (Center for Therapeutic Antibody Discovery at the Icahn School of Medicine at Mount Sinai, clone 1A7), and rabbit polyclonal anti-NEP (GeneTex, GTX125953). Primary antibodies were either detected using HRP-conjugated secondary anti-mouse (GE Healthcare, NA931V) and anti-rabbit (GE Healthcare, NA934V) antibodies, or fluorophore-conjugated, IRDye 680RD anti-mouse (LI-COR Biosciences, 926-68070) and IRDye 680RD anti-rabbit (LI-COR Biosciences, 926-32211) antibodies. The HRP/fluorophore-conjugated secondary antibodies are visualized using Immobilon Western Chemiluminescent HRP substrate kit (Millipore), or on the Odyssey imaging system (LI-COR Biosciences) according to the manufacturer’s instructions, respectively.

### Immunofluorescence staining

Cells were directly fixed in tissue culture plate using 90% methanol and permeabilized using PBS + 1%Triton X-100 (Sigma-Aldrich, 9036-19-5). The cells were blocked with PBS + 0.1% Tween-20 + 1% BSA for 1 h at room temperature. Cells were then incubated with primary antibodies, including mouse monoclonal anti-IAV NP (Center for Therapeutic Antibody Discovery at the Icahn School of Medicine at Mount Sinai, clone HT103), mouse monoclonal anti-IAV NS1 (Center for Therapeutic Antibody Discovery at the Icahn School of Medicine at Mount Sinai, clone 1A7), rabbit polyclonal anti-NEP (GeneTex, GTX125953) and rabbit monoclonal anti-GAPDH (Cell Signaling, 2118S), diluted 1:500 in the blocking buffer. The primary antibodies were visualized using fluorophore conjugated secondary antibodies, anti-Rabbit IgG Alexa 488 (Thermo Fisher A-21206) and anti-mouse IgG Alexa 594 (Thermo Fisher A-21203), diluted 1:1000 in the blocking buffer. The staining was visualized using EVOS M5000 imaging system (Thermo Fisher AMF5000).

### RNA sequencing

Total RNA from infected and mock treated cells was extracted using TRIzol (Thermo Fisher^®^) or the RNeasy Mini kit (QIAGEN^®^) according to the manufacturer’s instructions and treated with DNase I. RNA-seq libraries were prepared using the TruSeq RNA Library Prep Kit v2 (Illumina^®^), following manufacturer’s instructions. All sequencing runs were performed using an Illumina NextSeq 500 platform.

### Analysis of bulk RNA sequencing data

Sequencing reads were aligned to the human genome (hg19) using the STAR aligner (Dobin et al., 2013) followed by differential gene expression analysis by DESeq2 (Love et al., 2014), as implemented in the RNA Express application in BaseSpace (Illumina^®^). Heatmap plots of statistically significant differentially expressed genes (L2FC ≥2, adjusted *p*-value <0.05) were constructed using the heatmap.2 function form the ggplots package in R. Sequencing reads were also aligned to the IAV (A/Puerto Rico/8/34/Mount Sinai) genome reference using Bowtie2 (Langmead and Salzberg, 2012). Expression of NS1 transcripts was addressed by counting the total number of reads aligning to the non-overlapping NS1 portion in segment 8 (S8:27-514) using SAMtools (Li et al., 2009). The viral genome references for IAV (A/Puerto Rico/8/34/Mount Sinai) were: AF389122.1, AF389121.1, AF389120.1, AF389119.1, AF389118.1, AF389117.1, AF389116.1, AF389115.1. Defective viral genomes (DVGs) identification and quantification was performed using ViReMa (Routh and Johnson, 2014) following a previously described pipeline (Alnaji et al., 2019). Only strand congruent recombination events were quantified for IAV (to account for deletion DVGs). miRNA expression in NHBE cells was identified using the Small RNA application in BaseSpace (Illumina^®^) on previously published NHBE small RNA sequencing data (SRA: SRR5127216) (McCall et al., 2017). Sequencing data used in this manuscript is available on NCBI GEO under accession numbers GSE147507 (samples: GSM4462363, GSM4462364, GSM4462365, GSM4462366, GSM4462375, GSM4462376, GSM4462377, GSM4462378, GSM4462379, GSM4462380) and GSE243730.

### Gene set enrichment analysis

In order to select genes whose expression was differentially affected by IAV NS1 levels, we first ranked each gene based on its expression (TPM; transcripts per million) (i) in cells infected with IAV-ΔNS1 versus cells infected with IAV-WT or IAV-NS1-T (priority gene targets), or (ii) in cells infected with IAV-WT versus cells infected with IAV-ΔNS1 or IAV-NS1-T (secondary gene targets), using gene set enrichment analysis (GSEA) (Subramanian et al., 2005). The sign of the difference between the rank metric scores (signal to noise ratio) of both comparisons (any NS1—WT NS1) defined whether the gene was responsive to any NS1 (negative) or only to WT NS1 levels (positive). Only genes that had a rank metric score <−1 and were statistically significant differentially expressed (in any comparison or condition) were considered. Finally, the membership to a specific pattern (priority or secondary gene targets) was weighted considering the fold change in expression [L2FC; Log_2_(fold change)] of cells infected with IAV-WT (WT NS1) or IAV-NS1-T compared to IAV-ΔNS1 infections. The weighted list of selected genes for each pattern was used to identify enriched gene ontology (GO) annotations (biological process) using Enrichr (Kuleshov et al., 2016). Redundant GO annotations were reduced by eliminating annotations that had >75% overlap to another GO annotation using the reduce_overlap function from the GOplot package in R (Walter et al., 2015). Final annotations were visualized in R using custom scripts.

### RT-qPCR analysis

For qualitative analysis of host mRNA, total RNA was extracted using TRIzol (Thermo Fisher^®^) followed by RNA purification via Direct-zol-96 RNA Kit (VWR 76211) and treated with DNase I according to the manufacturer’s instructions. Extracted RNA was reverse transcribed using SuperScript IV (Thermo Fisher 18091050) with oligo-dTs. The cDNA was then diluted and used for qPCR analysis via PowerTrack SYBR Green master mix (Thermo Fisher. A46109). The qPCR was performed using BioRad CFX96 machine for 40 cycles, and the Ct values were used for relative fold change calculation via ∆∆Ct method. The primers used for qPCR are listed below:

**Table 1:** 

Target gene	Forward primer (5′-3′)	Reverse primer (5′-3′)
IL6	GGTCAGAAACCTGTCCACTG	CAAGAAATGATCTGGCTCTG
IFNB1	GTCAGAGTGGAAATCCTAAG	ACAGCATCTGCTGGTTGAAG
IFIT1	TCGGAGAAAGGCATTAGATC	GACCTTGTCTCACAGAGTTC
ISG15	ACAGCCATGGGCTGGGAC	TGATCTGCGCCTTCAGCTC
sXBP1	GCTGAGTCCGCAGCAGGT	CTGGGTCCAAGTTGTCCAGAAT
tXBP1	TGAAAAACAGAGTAGCAGCTCAGA	CCCAAGCGCTGTCTTAACTC
EDEM1	CAAGTGTGGGTACGCCACG	AAAGAAGCTCTCCATCCGGTC
HSPA5	TGTTCAACCAATTATCAGCAAACTC	TTCTGCTGTATCCTCTTCACCAGT
TUBA1A	GCCTGGACCACAAGTTTGAC	TGAAATTCTGGGAGCATGAC

### A549-DUAL reporter assay

A549-DUAL cells (Invivogen) are maintained in complete DMEM media supplemented with 10% FBS, pen-strep, L-glutamine, 100 μg/mL zeocin, 10 μg/mL blasticidin and 50 μg/mL normocin. The cells were seeded at 10^5^ cells/well into 96-well plates the day before treatment. The supernatant from NHBE treated with human IFN-β (100 U/mL), infected with WT or recombinant IAVs, or mock were harvested and filtered through Vivaspin column (MWCO 100 kDa) (Cytiva). The flowthrough supernatant containing the secreted cytokines are used to treat the seeded A549-DUAL cells (InvivoGen, a549d-nfis) at 100 μL per well for 12 h. The secreted alkaline phosphatase was quantified using QuanTI-Blue reagent (InvivoGen, rep-qbs), and the secreted luciferase was quantified via QuanTI-Luc reagent (InvivoGen, rep-qbs) on Synergy H4 plate reader (BioTek), following the manufacturers recommendation.

### Single cell RNA sequencing analysis

NHBE cells were infected with IAV-WT at MOI of 3 for 12 h. At 12 hpi, dead cells were washed off the plate, and the live cells were trypsinized and washed in PBS and centrifuged at 300 × *g* for 5 min. The cells were counted to ensure >90% viability and concentrated to 2.12 × 10^6^ cells per mL. A total of 10 μL of cells were hence loaded into PIPseq T20 3′ Single Cell RNA kit v4.0 [Fluent BioSciences, (Clark et al., 2023)] reaction tube to be processed, intended to capture 10,000 cells. Following the manufacturer’s protocol, the transcripts from cells were barcoded, and processed for Illumina sequencing on NextSeq2000 platform using P3-100 cycle kit. A total of 267 million reads were assigned to the sample. Initial read filtering, read counting and cell filtering were processed using PIPseeker v2.1 with custom genome containing both human and IAV/PR8/34 references.

Filtered count matrix was first processed through SoupX software to infer ambient contamination of viral RNAs (Young and Behjati, 2020). The SoupX-corrected count matrix was further filtered for low and high UMI counts and percentage of mitochondria reads. The filtered matrix was processed using Seurat v4.9 following the standard analysis pipeline to normalize read counts and generate UMAP layout (Hao et al., 2021). Specifically, SCT transformation was used to regress out the confounding effect of total UMI, number of features, % mitochondria and percent viral reads. To label infected cells, the log ratio of viral reads over total reads per cell was visualized on a density plot, and the threshold for infected cell was set to be the local minimum of the distribution (~0.3%, Supplementary Figure S5A). Within infected cells, the high/mid/low NS1 levels were defined based on the distribution of the log transformed NS1 expression level (Supplementary Figure S5B). Briefly, the NS1-low threshold is determined to be the local minimum of the distribution. To separate NS1-high and NS1-mid population, two normal distributions for NS1 expression were fitted to the remaining infected population via expectation-maximization algorithm (Benaglia et al., 2009), and the NS1-mid/high cutoff was set to be the local minimum of the two distributions. The calculation of average expression of the top priority and secondary target genes in the single cell data was done using Seurat function AddModuleScore. The complete analysis script is provided as an R markdown document at https://github.com/BlancoMeloLab/NS1_Priority.

## Results

### Modulation of influenza NS1 levels via insertion of host miRNA targeting sites

**Figure 1:**
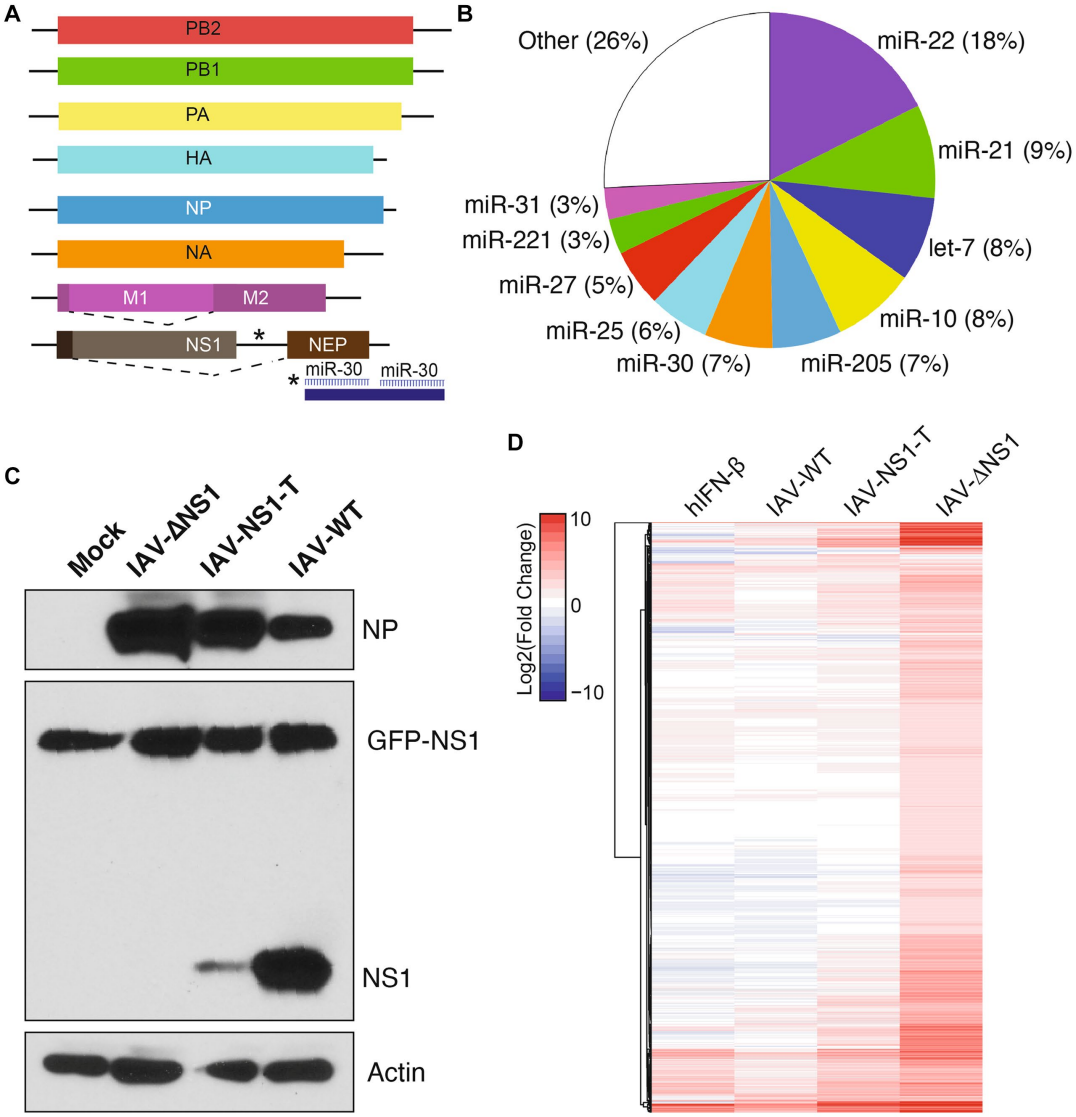
Insertion of host miRNA targeting sites in influenza genome modulates viral NS1 levels and host response during infection. **(A)** Diagram of recombinant IAV-NS1-T where two miR-30 target sites were introduced into the 3′ UTR of the NS1 ORF. **(B)** miRNA expression profile of NHBE cells at baseline. Values represent the percentage of total miRNA reads for each miRNA. Data from McCall et al. (2017). **(C)** NS1 protein levels in MDCK-NS1-GFP cells infected with wild-type IAV, IAV-∆NS1 or IAV-NS1-T for 12 h at a MOI of 1. MDCK-NS1-GFP cells constitutively express a NS1-GFP fusion protein. Whole-cell extracts were analyzed by Western blot for viral protein expression. **(D)** Heatmap depicting the transcriptional response of NHBE cells to IFN-β treatment or infection of IAV-WT, IAV-NS1-T, or IAV-ΔNS1 virus (*n* = 4, MOI = 3, 12 hpi). Values represent the Log_2_(fold change) expression (compared to mock-treated cells) for statistically significant differentially expressed genes in at least one condition. Values for IFN-β treatment represent an aggregate of cells treated for 4, 6 and 12 h compared to mock-treated (*n* = 2 for each time point).

We hypothesized that modulating IAV NS1 expression levels during infection would reveal the prioritization of their multiple host antagonistic functions. To achieve this, we utilized a previously described recombinant virus [lab adapted A/Puerto Rico/8/34 (H1N1)], in which the viral NS1 and NEP open reading frames are separated to enable the insertion of two miRNA targets (Chua et al., 2013) (Figure 1A), leading to the silencing of NS1 while sparing NEP expression. To achieve intermediate levels of NS1 silencing using miRNA target sites, we characterize the miRNA abundance in NHBE cells—using publicly available miRNA sequencing datasets—to search for a moderately expressed miRNA (McCall et al., 2017) (Figure 1B). We found that miR-30 is moderately expressed in NHBE cells, accounting for 7% of the total miRNA abundance. Thus, we incorporated two fully complementary target sites of miR-30 into the NS1-specific 3′UTR (IAV-NS1-T) (Figure 1A). We hence compared the NS1 expression levels during active infections via RNA-seq and western blotting, contrasting IAV-NS1-T with wildtype IAV (IAV-WT) and IAV-ΔNS1, a recombinant virus lacking NS1-specific open reading frame while expressing NEP (Garcia-Sastre et al., 1998). We confirmed that infection with IAV-NS1-T in MDCK-NS1 and NHBE cells showed that miR-30 drastically reduced NS1 expression to levels intermediate between IAV-ΔNS1 and IAV-WT viruses with minimum impact on NEP expression or overall level of infection (Figure 1C; Supplementary Figures S1, S2). In addition, by measuring the percentage of RNA-seq reads that span junctions in internal deletions over total viral reads, we validated that infection by the three influenza variants resulted in similar levels of viral transcripts and defective viral genomes (Supplementary Figure S2), a potent host immune response stimulant. Together, the three virus variants (IAV-WT, IAV-NS1-T and IAV-ΔNS1) enable us to compare the host response difference as a function of different viral NS1 levels.

### Modulation of viral NS1 levels reveals prioritization of host response antagonism

To compare host response during infections by the three virus variants, we performed RNA-seq followed by differential gene expression analyses to identify significantly upregulated genes compared to the mock condition (Figure 1D, *n* = 4 for each condition). To parse out which fraction of the host response is driven by type I interferons (i.e., ISGs), we also performed parallel sequencing analysis of NHBE treated with human IFN-β. Through comparison, we found the host response increased in magnitude and complexity with the decreasing level of viral NS1. Importantly, although viral NS1 is known to be a potent inhibitor of host ISG expression (Hale et al., 2008), IAV-NS1-T failed to restrict ISG expression with its intermediate level of NS1 when compared to IAV-WT, as shown in Figure 1D (Full transcriptomic analysis result is included in Supplementary Data 1). However, compared to IAV-ΔNS1, the intermediate level of NS1 still restricted a wide array of host genes, suggesting different host responses have different sensitivities to NS1 antagonism.

**Figure 2:**
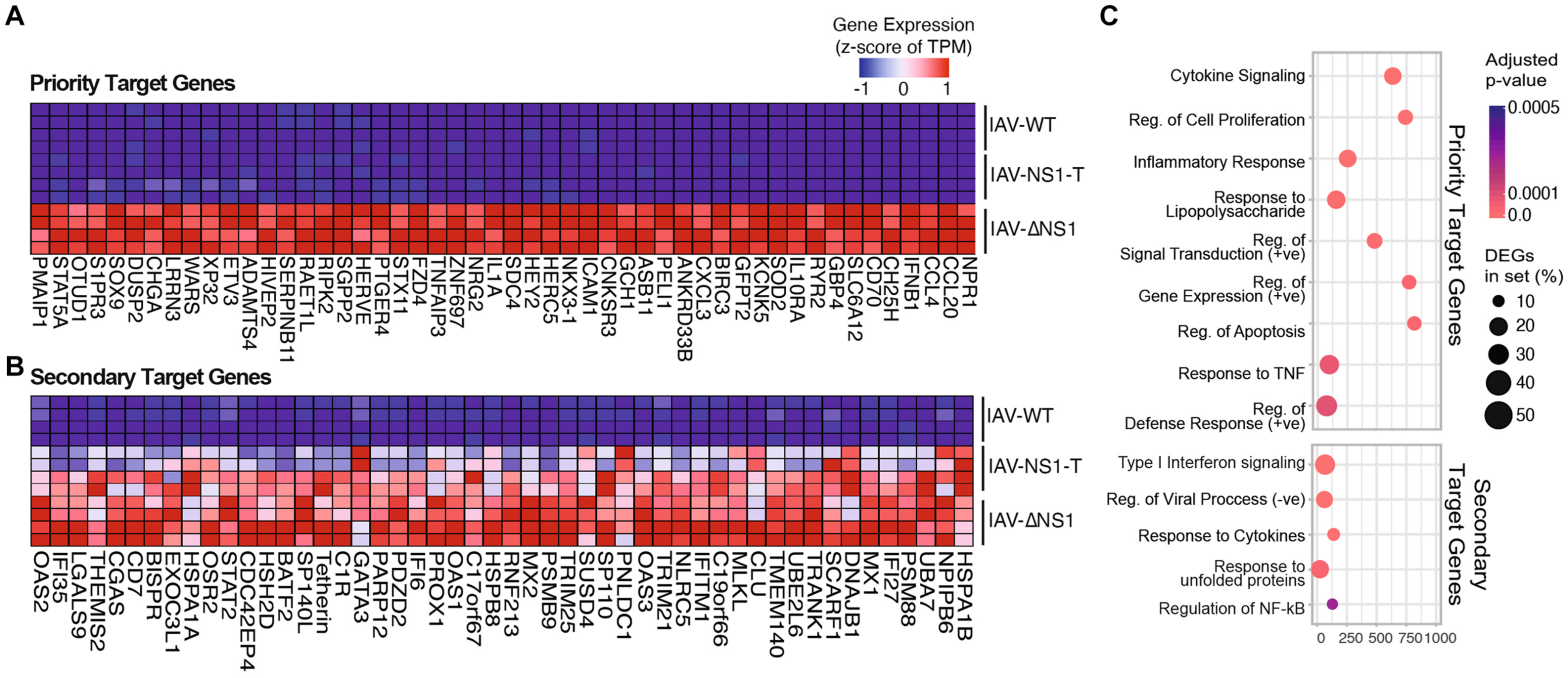
Modulation of NS1 level reveals prioritization of host response antagonism. **(A,B)** Heatmaps depicting the gene expression of the top 50 host gene markers (ranked by GSEA scores, see Methods) in NHBE cells infected with WT or recombinant IAVs, from comparisons between **(A)** NS1 negative (IAV-ΔNS1) vs. NS1 positive IAVs (IAV-WT, IAV-NS1-T). **(B)** IAV-WT vs. mutant NS1 IAVs (IAV-NS1-T, IAV-ΔNS1). **(C)** Top enriched GO terms in cells infected with NS1 negative IAV compared to NS1 positive IAVs (priority target genes, upper panel), or WT-IAV compared to mutant NS1 IAVs (secondary target genes, lower panel). The color of the dots represents the adjusted *p*-value for each enriched gene set. The size of the dots represents the percentage of differentially expressed genes enriched in the complete gene set.

To better determine how the antagonistic potency of NS1 is dependent on its abundance, we carried out pairwise ranked analysis using gene set enrichment analysis (GSEA). That is, differentially expressed genes between IAV-NS1-T and IAV-ΔNS1 would indicate host responses that can be efficiently blocked with just moderate levels of NS1, i.e., priority target genes (Figure 2A; Supplementary Figure S3A and Supplementary Data 2). In contrast, host responses that are similar between IAV-NS1-T and IAV-ΔNS1 as compared to IAV-WT would indicate pathways that require high levels of NS1 to achieve inhibition, i.e., secondary target genes (Figure 2B; Supplementary Figure S3B and Supplementary Data 2). Based on this logic, we first examined genes in which gene expression profiles for IAV-WT and IAV-NS1-T were comparable and distinct from IAV-ΔNS1 (Figure 2A; Supplementary Figure S3A). Enriched biological processes from this subset of host transcripts are characterized by cytokine induction and pro-inflammatory responses (Figure 2C upper panel, and Supplementary Data 2). In contrast, when comparing differentially expressed genes between IAV-WT and conditions in which NS1 was absent or limiting (IAV-NS1-T and IAV-ΔNS1), we observed the expression of several ISGs in addition to chaperones involved in the unfolded proteins response (UPR) (Figures 2B,C lower panel, and Supplementary Data 2). Together, these findings suggest that the viral NS1 protein prioritizes antagonizing the initial viral sensing and cytokine signaling pathways over the downstream ISG and UPR pathways, highlighting a ranking in host antagonism that is tailored to the viral replication kinetics and progressive events in the viral life cycle.

### Functional validations of host antagonism prioritization by influenza NS1

**Figure 3:**
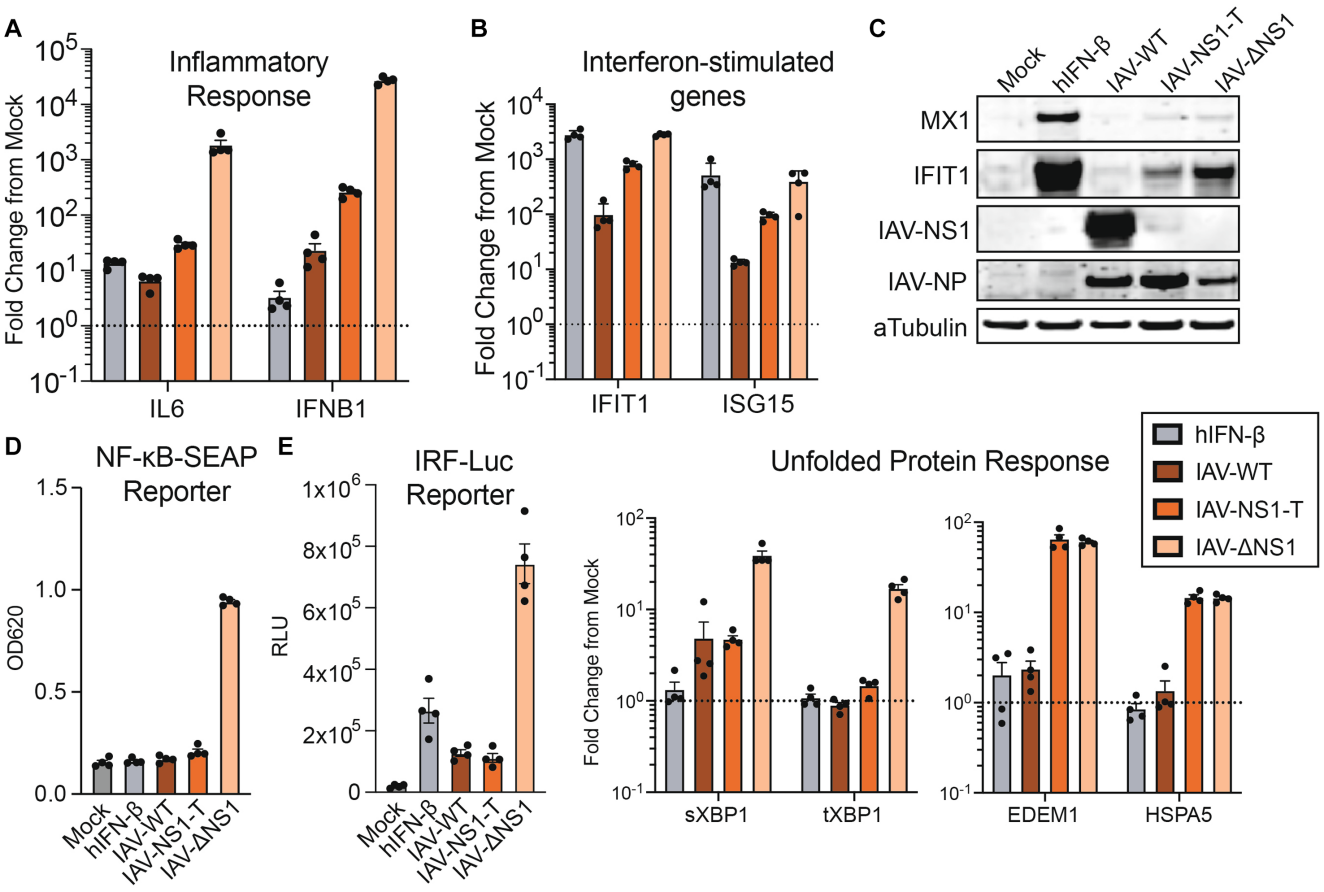
The activation of IFN response is sensitive to NS1 antagonism, while the silencing of unfolded protein response requires the highest amount of NS1. **(A,B)** RT-qPCR quantification of relative fold changes of signature gene inductions representing diverse responses in NHBE cells infected with WT or recombinant IAVs, compared to mock. TUBA1A was used as the internal control, and primers targeting indicated signature genes are used to profile the activation of IFN response **(A)** and ISGs **(B)**. **(C)** Western blot validation of the ISG and viral protein expressions in NHBE treated with human IFN-β (100 U/mL), infected with WT or recombinant IAVs, or mock. **(D)** Quantifications of pro-inflammatory cytokines and IFN secretion by NHBE cells treated with human IFN-β (100 U/mL), infected with WT or recombinant IAVs, or mock. Filtered supernatant from the infected samples were used to treat reporter cell line A549-DUAL. The relative activation of NF-kB or ISRE by each supernatant were quantified via alkaline phosphatase or luciferase assay, respectively. **(E)** RT-qPCR measurement of transcript fold change involved in the UPR, compared to mock. Experiments were performed as in **D** and **E**.

To validate our transcriptomic findings, we profiled the host response to infection at the RNA, protein, and functional levels. We observed a NS1 dosage-dependent host response for genes involved in the inflammatory response (IL-6, IFNB1) and interferon stimulation (IFIT1, MX1, ISG15), with the IAV-NS1-T infection generating an intermediate phenotype at both the transcript (Figures 3A,B) and at the protein levels (Figure 3C). Consistently, we found that intermediate levels of NS1 were sufficient to block the release of pro-inflammatory cytokines in reporter cells, where the supernatant from both IAV-WT and IAV-NS1-T infections failed to induce detectable activity for both NF-kB-dependent alkaline phosphatase and IFN-dependent luciferase reporters (Figure 3D). Interestingly, antagonizing host UPR appears to require the highest level of NS1. Although a low level of XBP1 splicing due to ER stress is observed in both IAV-WT and IAV-NS-T infection (Figure 3E left), silencing of the UPR activation—marked by the transcriptions of XBP1-regulated genes EDEM1 and HSPA5 (and other chaperone proteins highlighted in Figure 2B) (Oslowski and Urano, 2011)—is only observed during IAV-WT infection (Figure 3E right). This is consistent with the understanding that NS1-mediated UPR silencing is achieved at the transcriptional level, by interrupting pre-mRNA processing (Mazel-Sanchez et al., 2021), which would not affect ER stress-dependent splicing of XBP1 (Yoshida et al., 2001). Furthermore, these results draw a striking parallel to a time course of IAV-ΔNS1 HD infection in NHBE cells, where a comparable dynamic cascade of host transcription can be observed leading to the activation of ISG and UPR at later time points (Supplementary Figure S4 and Supplementary Data 3). These results are consistent with the understanding that the host response intensifies as viral products (i.e., RNA, protein) accumulate over the course of the infection (Nilsson-Payant et al., 2021). As such, subsequent stages in the viral life cycle progressively demand increasing levels of NS1 to inhibit relevant host transcriptional programs. Altogether, our data highlights that the inhibition of host responses by IAV NS1 is tailored to the virus replication kinetics, with the initial viral sensing and cytokine production being the most sensitive to NS1 expression, and the UPR—the ER stress response driven by the high accumulation of viral proteins at later stages of the viral life cycle—requiring the highest levels of NS1.

### Heterogeneity in host response is shaped by differences in viral NS1 levels in single cells

**Figure 4:**
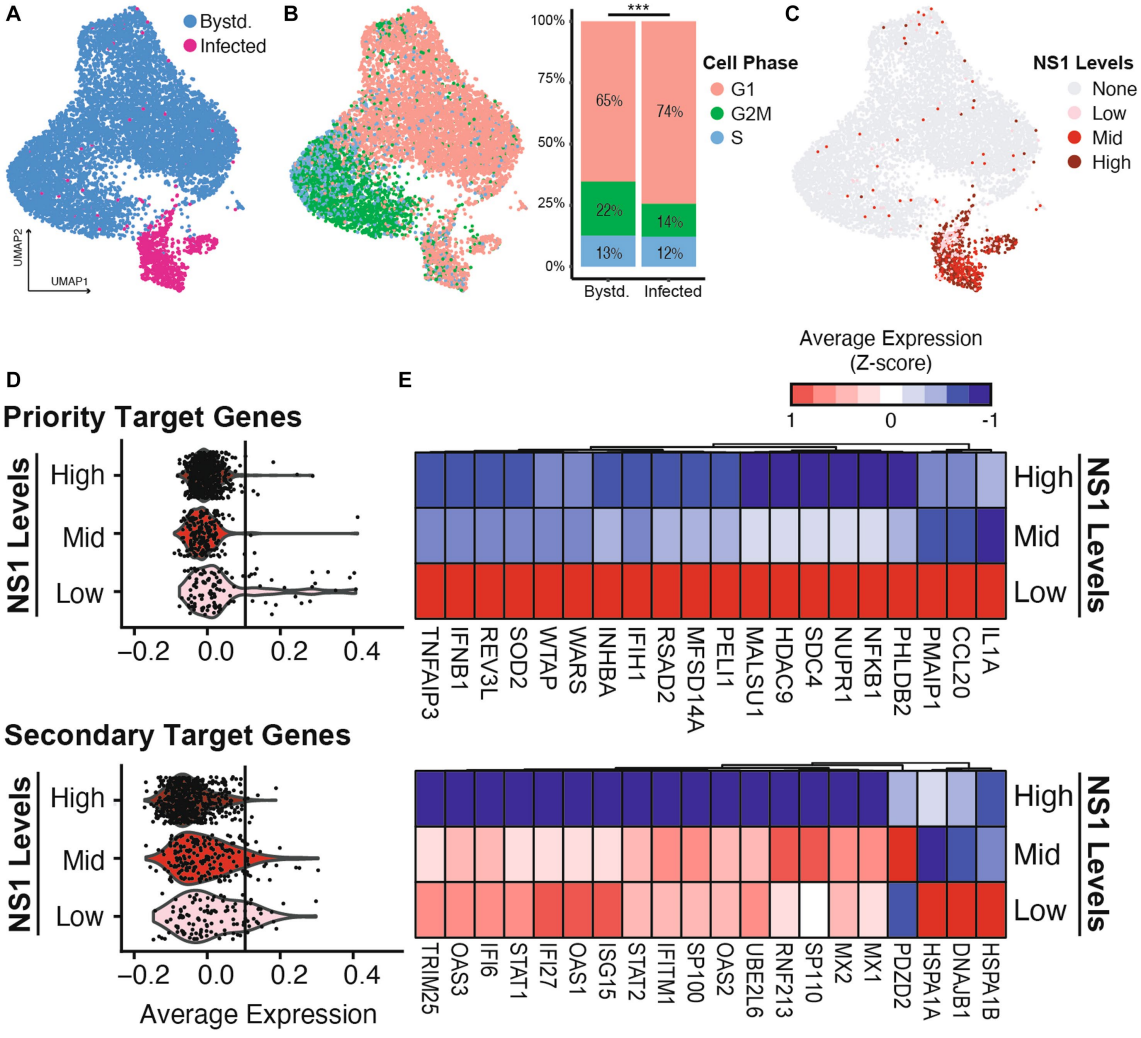
Host response heterogeneity during IAV-WT infection is shaped by the differential levels of viral NS1. NHBE cells were infected with wild type IAV at MOI of 3 for 12 h before being processed for scRNA sequencing. **(A)** Uniform manifold approximation and projection (UMAP) representation of the scRNAseq dataset with infected (red) and bystander (blue) cells highlighted (see Methods and Supplementary Figure S5A). **(B)** Using the same UMAP layout, cell cycle estimation is color-coded (G1: pink, G2M: green, S: blue) based on the expression of marker genes. The distribution of cell phase in bystander and infected population were compared. ^***^p-value <0.001, chi-squared test. **(C)** Based on the distribution of IAV NS expression, the infected cells are separated into low, mid and high bins (Supplementary Figure S5B). **(D)** Violin plots illustrating the average expressions of priority and secondary target genes in infected cells separated by different NS1 levels. The average expression of the top 100 genes from each category (Figure 2A) was used to calculate each module gene score. To highlight the differences between expressions in different NS1 levels, vertical lines are included to represent the 99% quantile of the average expression by NS1-high cells in each category. **(E)** Heatmap showing the average expressions of the top 20 detectable genes from each host response category, by infected cell separated by different NS1 levels.

The bulk analyses above demonstrated differences in the averaged host responses across three different infection conditions. However, previous studies indicate a heterogeneity in the viral content and host response during any infection (Russell et al., 2018; Kupke et al., 2020). We thus sought to investigate whether the host antagonism prioritization can also be demonstrated by cell-to-cell NS1 expression heterogeneity during wild-type IAV infection. We employed single cell 3′ RNA-seq technology to characterize both viral transcript abundance and host response in NHBE infected with IAV-WT (MOI = 3, 12 hpi). At high MOI infection, a fraction of cells supported active viral replication, marked by having significant number of viral reads over total reads (Figure 4A; Supplementary Figure S4A, 9.5% infected out of 9,066 cells, see Methods). This percentage is consistent with previous reports and is lower than the theoretical percentage given the MOI of 3 (i.e., ( $1-e^{-3}$ ) × 100% = 95%), as dead cells were excluded during the library preparation and data filtering steps (Russell et al., 2018). Compared to the bystander cells, more infected cells underwent cell cycle arrest, as indicated by a higher percentage of cells at G1 phase (Figure 4B, 74% infected vs. 65% bystander, *p*-value = 1.15 × 10^−8^, Chi-squared test). Importantly, we observed high level of heterogeneity in viral NS1 expression levels in infected cells, even after normalizing read counts for ambient RNA and percent viral reads (Figure 4C; Supplementary Figure S4B, see Methods). Based on the distribution of NS1 levels, we further separate infected cells into NS1 high, mid, and low levels (represented by 541, 199, and 118 cells, respectively, Supplementary Figure S5B). This enabled the examination of host response differences in relationship to the viral NS1 abundance within individual infected cells.

Focusing on the two categories of host response that are facing differential antagonism prioritization by viral NS1 (Figures 2A,B), we observed that the priority target genes (cytokines and inflammatory response) are only upregulated in infected cells with the lowest amount of NS1 (Figures 4D,E upper panels), while the infected cells with intermediate amount of viral NS1 are still able to induce the expression of the secondary target genes (ISGs and some UPR genes, Figures 4D,E lower panels). Importantly, the priority target genes, including type I IFN and pro-inflammatory cytokines, are only expressed in infected cells with low level of NS1 but high level of overall viral load (Supplementary Figure S5C). On the other hand, the activation of ISG expression is independent of the overall viral load, and their inhibition is limited to the highest level of NS1 (Supplementary Figure S5C). This suggests ISG expression in infected cells is likely driven by paracrine signaling from secreted IFNs and countered by intracellular NS1, resulting in a linear dosage-dependent repression. On the other hand, contrasting to the bulk RNA-seq analysis, where the intermediate NS1 level during IAV-NS1-T infection still permitted UPR activation (Figure 2B), infection by IAV-WT with an intermediate level of NS1 (NS1-mid) did not robustly activate UPR (e.g., the lack of upregulations of heat shock proteins HSPA1A, HSPA1B, or DNAJB1 in Figure 4E). This is likely a combined result of difference in the amount of viral replication between experimental conditions, as well as the subtle differences in the exact amount of viral NS1 in infected cells (i.e., IAV-WT NS1-mid cell population may represent a higher NS1 level than the level achieved by IAV-NS1-T). Together, using single cell transcriptomics, we further validated that the heterogeneity in host response during wild-type IAV infection is a result of variations in NS1 levels in infected cells.

## Discussion

To overcome the inherent biological conflict between replication and host recognition, IAV uses NS1, a potent immune antagonist, to suppresses an expansive set of host immune functions. To dissect which host transcriptional programs were more sensitive to NS1 suppression, we modulated the levels of NS1 in the context of a functional infection, through direct miRNA targeting of NS1 transcripts (Figure 1). From bulk transcriptomic analysis, we uncovered two groups of host response that are differentially antagonized by NS1: IFN and pro-inflammatory cytokine expressions are effectively silenced with moderate amount of NS1 (priority targets), while a higher level of NS1 is required to suppress ISG and UPR expressions (secondary targets) (Figures 2, 3). Such prioritization further shaped the heterogeneity in host response during IAV infection at the single cell level, providing fine details of the IAV NS1 antagonism (Figure 4).

The observed prioritization of host immune antagonism by NS1 is likely the combined results of (1) the adaptation to viral replication kinetics and (2) the inherent differences in mechanism and affinity against host immune regulatory effectors. First, in concert with IAV life cycle, a modest level of NS1 was sufficient to inhibit virus detection and subsequent expression of IFNs and other pro-inflammatory cytokines, as these activations happen at the early stages of viral replication where viral transcripts are limited (Phan et al., 2021). As viral products accumulate in the cells, IFN secretion becomes unavoidable in cells lacking NS1, and cells with higher levels of NS1 were able to prevent the induction of ISG from paracrine signaling. Finally, inhibition of chaperones related to the UPR requires the highest expression of NS1, as structural viral proteins inundate the endoplasmic reticulum (Frabutt et al., 2018). Indeed, previous study by Mazel-Sanchez et al. (2021) suggests the accumulation of viral neuraminidase (NA) is the main contributor of ER stress, and such ER stress is modulated by wild-type level viral NS1 to prevent the full activation of UPR. Our findings further demonstrate the dynamics between the degree of ER stress and the dosage of NS1, indicating that the UPR is only antagonized by the highest level of NS1 (that of IAV-WT), despite unavoidable ER stress triggered by all three IAV constructs (i.e., IAV-WT, IAV-NS1-T and IAV-ΔNS1). In fact, it was indicated that IAV uses the low level of ER stress to its advantage to tolerate mutations in the viral surface protein NA and HA (Phillips et al., 2018). Such adaptation could further allow the virus to avoid adaptive immune recognition, and hence, evolutionarily, UPR antagonism has a lower priority.

In addition, the different sensitivities to NS1 antagonism can also be driven by the underlying mechanisms of viral-host interaction. NS1 contains both nuclear export and localization signals, contributing to its expansive location during viral replication and allowing it to interact with diverse cellular targets. Regulation of IFN induction by NS1 is achieved through multiple direct interactions, including its binding with RIG-I or its upstream activators, TRIM25 and Riplet (Gack et al., 2008; Rajsbaum et al., 2012; Jureka et al., 2020), as well as the interaction with IKK, which is required for NFκB activation (Gao et al., 2012). On the other hand, silencing of ISG expression and ER stress response is mainly achieved at the transcriptional level, through the disruption of pre-mRNA processing by binding to CPSF30 and the inhibition of host transcription termination (Nemeroff et al., 1998; Heinz et al., 2018; Mazel-Sanchez et al., 2021). Surprisingly, previous studies have shown that the NS1 from IAV/PR8/34 failed to interact with CPSF30 compared to other strains (Kochs et al., 2007; Nogales et al., 2019), but we still observed PR8 NS1 dosage-dependent inhibition of ISG expression and UPR at the bulk and single-cell levels. This further highlights IAV NS1 may directly inhibit ISG and UPR activation independent of its ability to inhibit IFN signaling or its interaction with CPSF30. In fact, a recent study found that IAV PR8 infection induces massive changes in host alternatively splicing by interfering with host splicing factor hnRNP K (Thompson et al., 2020), demonstrating additional host antagonistic strategies. Nevertheless, the prioritization against IFN induction over ISG and UPR activation is not only evident through our analyses but also at the conceptual levels—IAV NS1 interacts with many more upstream effectors of IFN induction than other pathways. Furthermore, such distinction could also be explained by the differences in relative abundance of nuclear vs. cytoplasmic NS1, as well as the different affinity to diverse host immune effectors. Follow-up studies should take advantage of the presented recombinant virus system to fine tune viral protein levels, guided by existing cell-line specific miRNAomic profiles: the viral protein level can be fine-tuned by introducing alternative miRNA targeting sites, or by increasing/decreasing the copy number of targeting sites (Langlois et al., 2013). This will enable the investigation of dosage-dependent viral-host interactions at a finer resolution.

Finally, our single cell analysis further highlighted that the observed difference in host response sensitivity is a combined effect of both variation in the ‘frequency” of NS1 (percentage of infected cells that are NS1 positive) and the overall NS1 “abundance” (the level of NS1 within individual cells). In the case of IFN and pro-inflammatory cytokine inductions, both the highest amount of viral transcription and the lowest NS1 level are required, consistent with recent findings by Vicary et al. (2023). During wild type IAV infection, this is an extreme rare event (Supplementary Figure S5C). The greater cytokine induction observed in IAV-ΔNS1 infection is likely due to the increased levels of viral replication without NS1 (i.e., increased “frequency” of NS1-negative infected cells), leading to more IFN-producing cells. On the other hand, we observed NS1 dosage-dependent suppression of host ISG and UPR expression that is independent of the amount of viral transcription. This suggests the inhibition of these response is highly dynamic—at the intermediate level, NS1 failed to fully silence these response, likely due to an unsaturated inhibition of host transcription. This intricate relationship is further complicated by differences in NS1-dependent host immune suppression between different IAV strains, follow-up studies should focus on comparing the strain differences in the host-specificity, frequency, and abundance of the NS1 in relationship to the magnitude of host response suppression (Sun et al., 2020).

Together, our data highlights the prioritization of the multiple host immune antagonisms by NS1. Such prioritization is likely an adaptation to the viral replication kinetics in relationship to host immune recognition of viral products, and a reflection of the variation in the selective pressure by different host immune pathways. Our results highlighted the distribution of IAV NS1 as the root cause of the heterogenous host response in a relevant primary cell model. These findings are fundamental to expose the weak links in IAV host adaptation to inspire future antiviral strategies.

## Data availability statement

The raw sequencing data presented in this study can be found in NCBI GEO repositories (www.ncbi.nlm.nih.gov/geo) under the accession numbers: GSE147507 and GSE243730. All other original contributions presented in the study are included as Supplementary material. Further inquiries can be directed to the corresponding author.

## Ethics statement

Ethical approval was not required for the studies on humans in accordance with the local legislation and institutional requirements because only commercially available established cell lines were used.

## Author contributions

QY: Methodology, Writing – review & editing, Data curation, Formal analysis, Investigation, Software, Validation, Visualization, Writing – original draft. AE: Formal analysis, Methodology, Writing – review & editing. MP: Methodology, Writing – review & editing. TL: Methodology, Writing – review & editing. BN-P: Conceptualization, Formal analysis, Methodology, Writing – review & editing. DB-M: Conceptualization, Funding acquisition, Methodology, Project administration, Supervision, Writing – review & editing.
