# Supplementary figures and images for: Modulation of Influenza A virus NS1 expression reveals prioritization of host response antagonism at single-cell resolution
Source: Front Microbiol. 2023 Oct 9;14:1267078. doi: 10.3389/fmicb.2023.1267078 (PMC10590924; doi:10.3389/fmicb.2023.1267078)

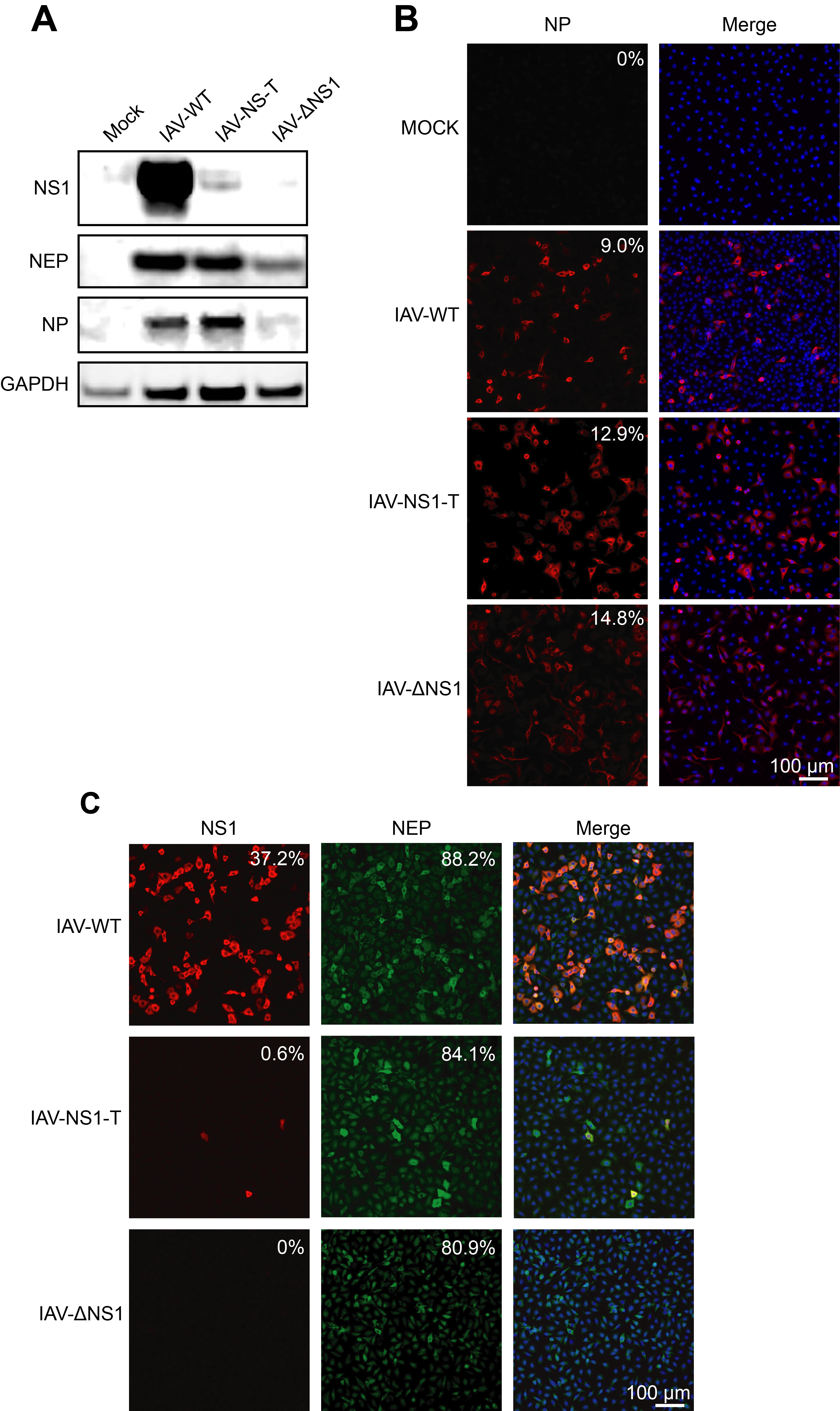

Supplement: Supplementary file 4 [file Image_1.JPEG]

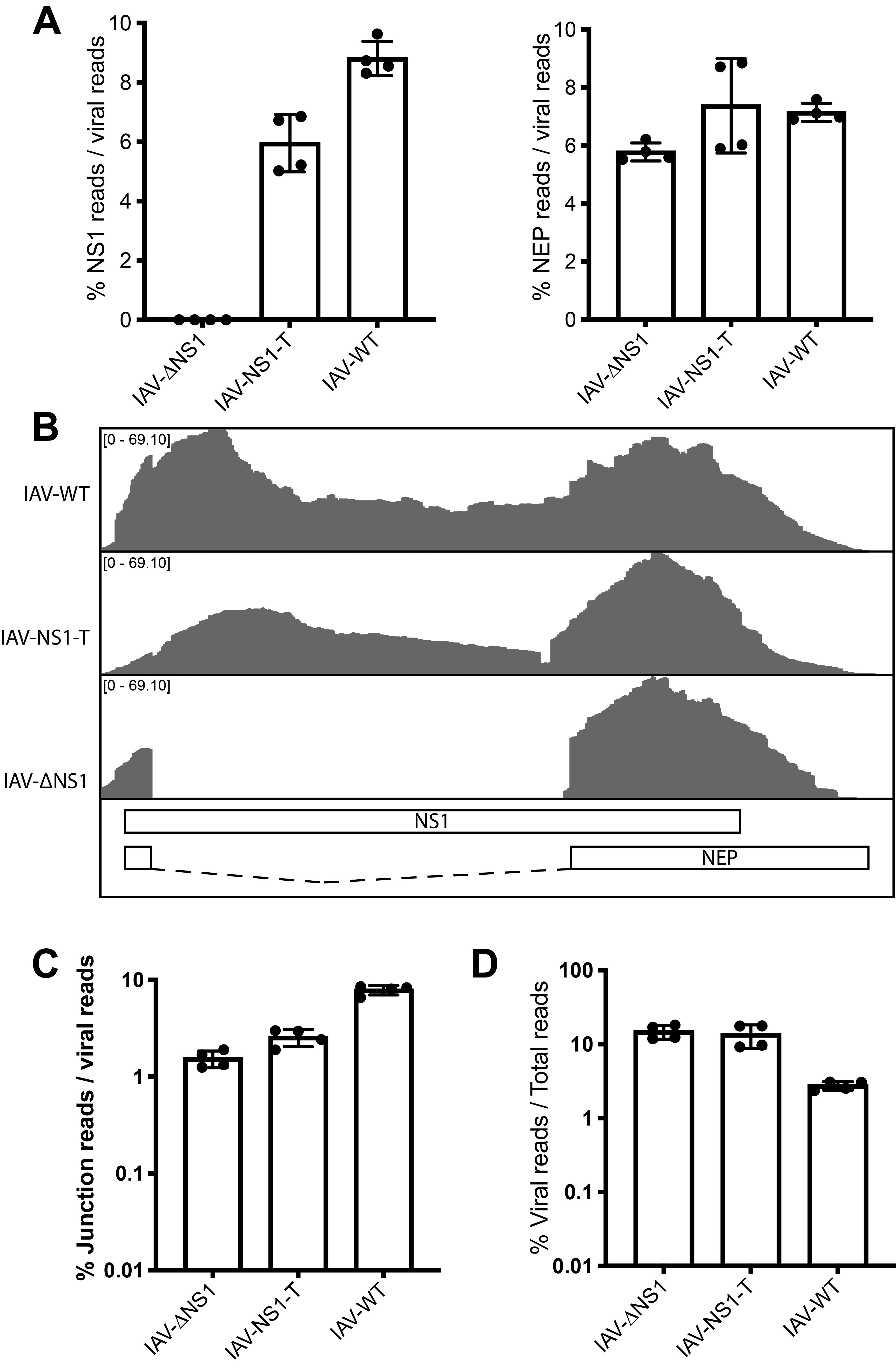

Supplement: Supplementary file 5 [file Image_2.JPEG]

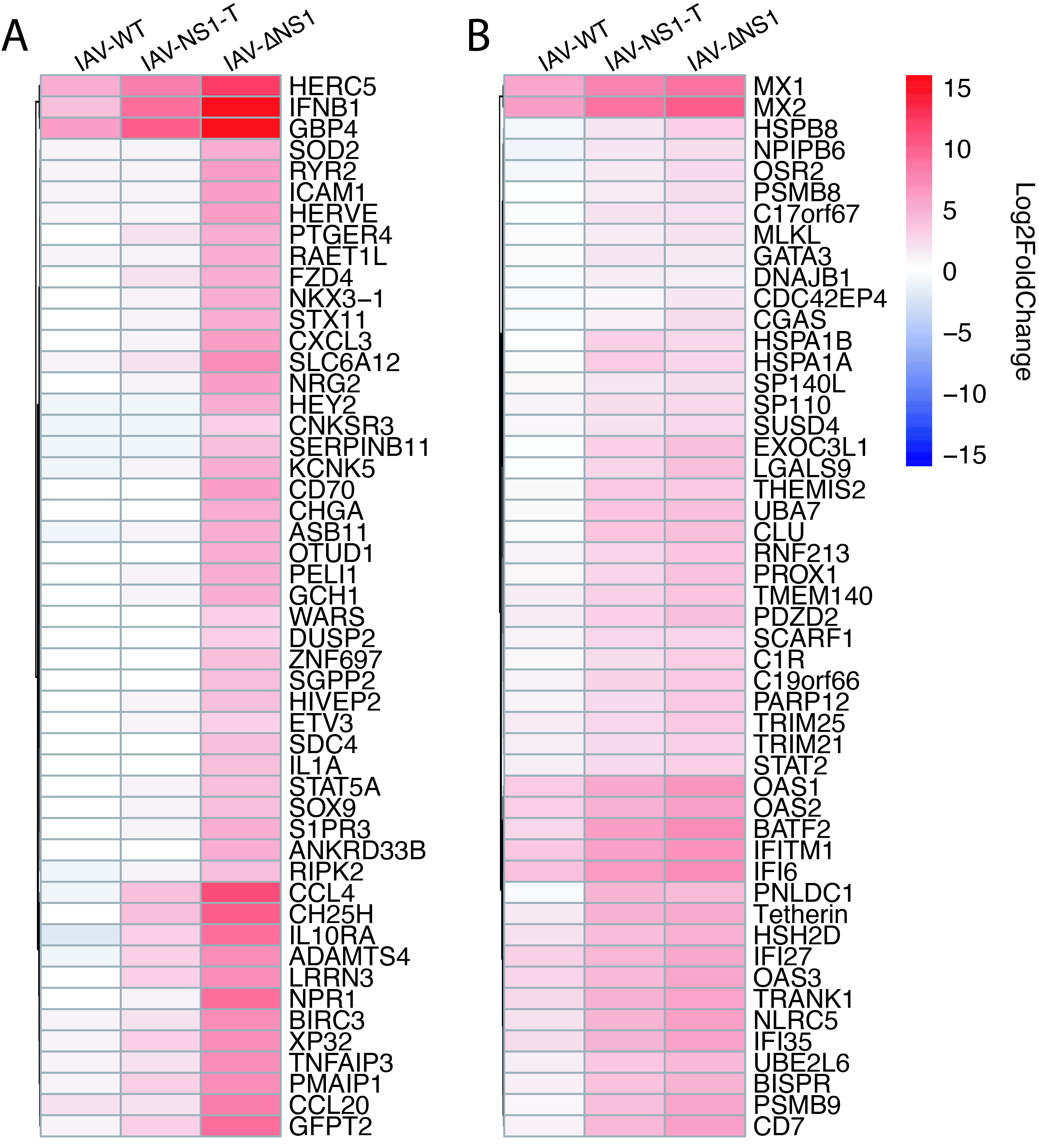

Supplement: Supplementary file 6 [file Image_3.JPEG]

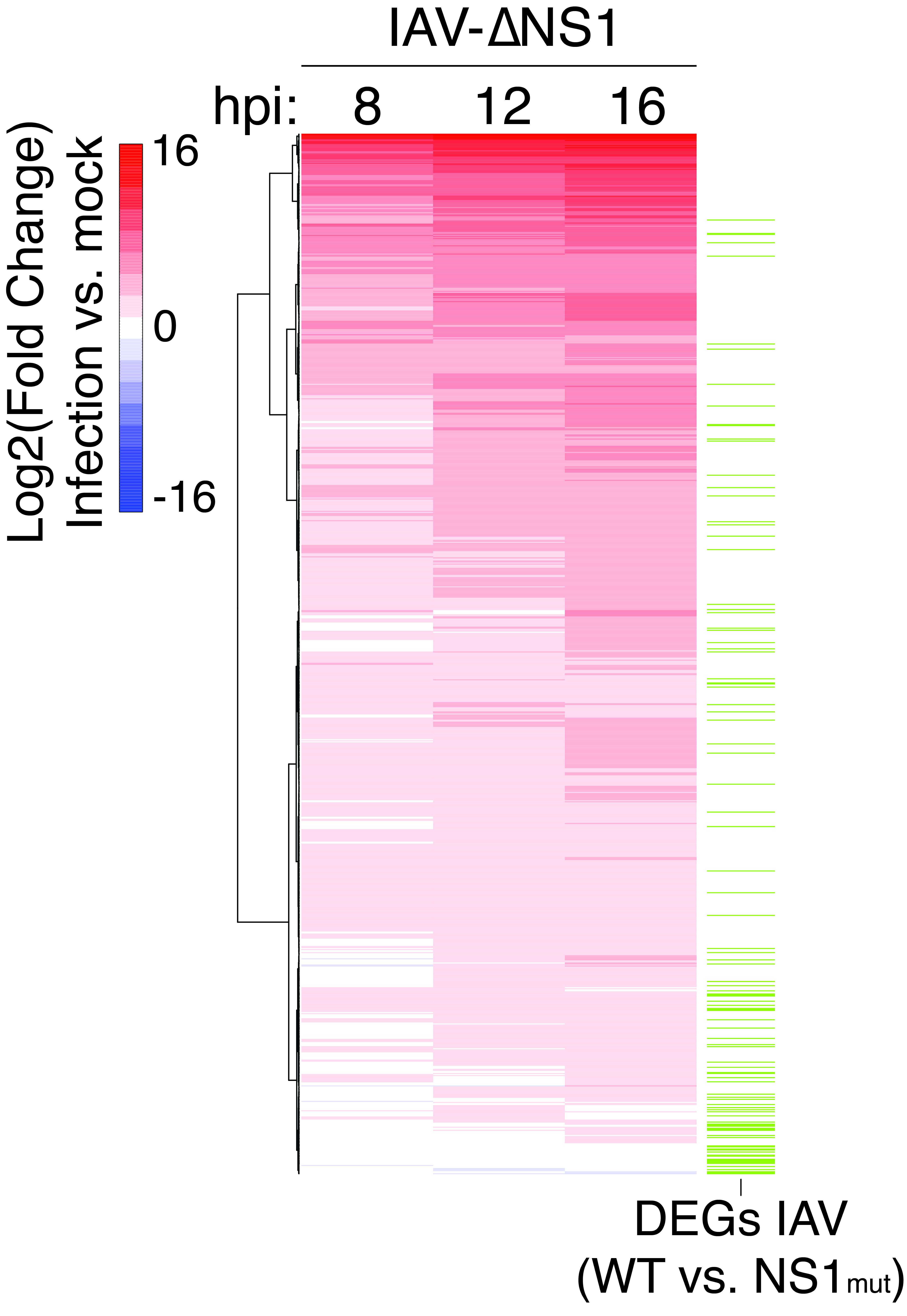

Supplement: Supplementary file 7 [file Image_4.JPEG]

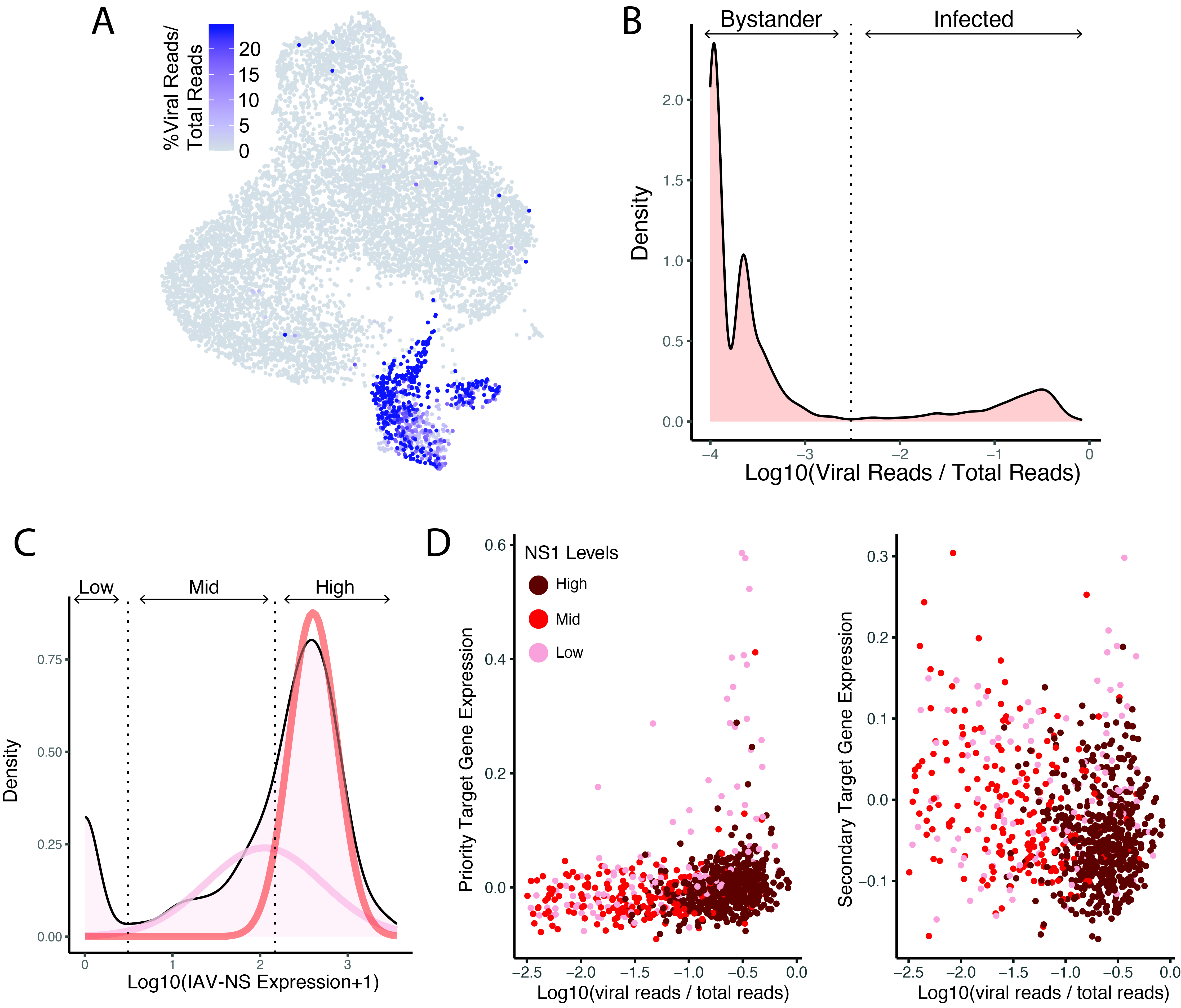

Supplement: Supplementary file 8 [file Image_5.JPEG]
